# Assessment of Burden in Caregivers of Patients Undergoing Hemodialysis and Peritoneal Dialysis: A Cross-Sectional Study in Riyadh, Saudi Arabia

**DOI:** 10.7759/cureus.52513

**Published:** 2024-01-18

**Authors:** Abdullah A Alaryni, Fadel Alrowaie, Abdullah Alghamdi, Razan Alabdullah, Raneem A Alnutaifi, Renad Alajlan, Raed A Alnutaifi, Amani Aldakheelallah, Alanoud Alshabanat, Abdullah S Bin Shulhub, Othillah M Moazin, Rayan Qutob, Enad Alsolami, Osamah A Hakami

**Affiliations:** 1 Internal Medicine, Imam Mohammad Ibn Saud Islamic University, Riyadh, SAU; 2 Nephrology, King Fahad Medical City, Riyadh, SAU; 3 College of Medicine, Imam Mohammad Ibn Saud Islamic University, Riyadh, SAU; 4 College of Medicine, King Saud University Medical City, Riyadh, SAU; 5 Internal Medicine, University of Jeddah, Jeddah, SAU; 6 Internal Medicine, King Abdullah Medical City, Makkah, SAU

**Keywords:** zarit burden interview, peritoneal dialysis, hemodialysis, caregiver burden, caregivers

## Abstract

A caregiver attends to the needs or concerns of someone limited by disease, injury, or disability to enhance the patient's quality of life, which can be assessed in three areas: social, physical, and psychological. This cross-sectional study assessed the extent of burden experienced by the caregivers of patients undergoing hemodialysis (HD) and peritoneal dialysis (PD) therapy in King Fahad Medical City in Riyadh, Saudi Arabia. The Zarit Burden Interview Arabic Abridged version (ZBI-A) was used to assess the level of burden experienced by caregivers. The data was collected and examined by professionals using the SPSS version 23. Based on the data of 50 participants, a mean ZBI-12 score of 12.22 ± 7.2 was reported. According to the ZBI scale, "No to mild burden," "Mild to moderate burden," and "High burden" were reported as 46% (n = 23), 38% (n = 19), and 16% (n = 8) of participants, respectively. The internal consistency of the ZBI-12 scale, assessed using Cronbach's alpha, was 0.664, indicating a satisfactory level of internal consistency. It was determined that caregivers of individuals undergoing PD and HD encounter different degrees of burden, with a significant proportion of caregivers experiencing a substantial burden.

## Introduction

End-stage kidney disease (ESKD) is a medical condition characterized by an irreversible deterioration in kidney function, reaching a severity that can lead to fatality without Renal Replacement Therapy. The global prevalence of ESRD is approximately 1,500 cases per 1 million individuals [1]. Saudi Arabia currently has 19,659 patients undergoing renal replacement therapy (RRT), with 18,270 of them receiving hemodialysis (HD) and the remaining 1,389 undergoing peritoneal dialysis (PD) [1,2]. Most individuals undergoing dialysis are aged 26 to 65, although they include elderly and frail patients. The provision of care for ESRD patients at home often requires the involvement of caregivers [1,2]. With the anticipation of providing medical attention to an aging population suffering from chronic ailments, physicians must evaluate the level of burden experienced by caregivers and gain a thorough understanding of their requirements. These aspects have progressively become crucial elements of complete clinical care [3]. 

HD plays a vital role as a treatment method in removing surplus fluids and metabolic waste substances in cases where renal function is impaired [4]. Patients dependent on HD require regular visits to specialized dialysis centers, which can lead to significant time and tiredness challenges. Moreover, individuals undergoing HD are prone to experiencing many sequelae, most notably anemia, skeletal abnormalities, and cardiovascular morbidity [5]. PD removes excess fluids and waste via the peritoneal membrane, making it convenient for patients and carers. PD patients often develop peritonitis, hernias, and catheter-related infections [5]. 

A caregiver is an individual who assists and supports those experiencing limitations resulting from illness, injury, or disability to enhance the patient's overall quality of life. This evaluation of quality of life encompasses three key domains: social, physical, and psychological. The domains under consideration are subject to the influence of individuals diagnosed with ESRD and undergoing dialysis [6]. The term "caregiver burden" encompasses the various challenges experienced by individuals providing care for a family member, typically in the context of their medical condition. These challenges can manifest in physical, financial, and psychosocial forms [7]. 

Carer burden includes physical, emotional, and financial challenges. HD carers transport patients to and from dialysis centers and manage their diets and medications [8]. However, PD carers must support dialysis, monitor food and pharmacological programs, and check for infections. Research showed PD carers had a lower carer burden than HD carers [6]. 

Dialysis is a life-saving measure for individuals suffering from chronic renal failure, albeit inducing substantial alterations to their lifestyles. HD has been observed to detrimentally impact patients' energy levels, impairing their capacity to engage in occupational tasks and perform routine daily activities. Consequently, this disruption to the normal functioning of patients and their caregivers can significantly compromise their quality of life [9,10]. The psychological burden of providing care and its correlation with adverse health effects have been extensively studied in caregivers from multiple countries with different cultural backgrounds [11,12]. An increase in caregiving responsibilities and a decline in overall well-being can lead to various complications, including the onset of depressive symptoms. A notable correlation exists between enhanced care burden and diminished quality of care provided by caregivers because the burden can significantly harm individuals. The stress levels experienced by caregivers of individuals with chronic illnesses constitute a significant health concern [4]. Cantekin conducted a descriptive study to examine the burden experienced by primary caregivers of patients undergoing dialysis. The caregiver burden for HD patients was reported to be 13%, whereas the PD group experienced a higher burden of 35% [13]. A cross-sectional study conducted in Indonesia examined the caregiver burden among 40 individuals providing care for a family member undergoing dialysis. The study assessed the potential relationship between caregiver burden and various factors, including gender and knowledge level. The findings indicated a statistically significant association between gender and knowledge level with high levels of caregiver burden. However, no significant associations were observed between caregiver burden and factors such as age, education level, and treatment duration [14]. In a recent study conducted in 2020, a sample of 170 individuals who were family members of PD patients was examined. The study revealed that 60% of these individuals experienced a mild to moderate level of burden, while 18.2% reported a moderate to severe level of burden. These findings were determined using various assessment tools [15]. 

The quality of life for caregivers of dialysis patients is negatively impacted compared to individuals of the same age and sex in society. These caregivers bear a significant burden of maintaining the patients' well-being and face an elevated risk of depression, particularly when social support is inadequate [16]. Hence, it is necessary to conduct a study to assess the extent of caregiver burden experienced by the caregivers of patients undergoing dialysis. 

Conducting a comparative analysis of the caregiver burden experienced by patients undergoing HD and PD is essential to optimize care provision, improve carers' overall well-being, allocate resources effectively, and foster advances in research and innovation within renal care. 

The objective of this study was to perform a cross-sectional investigation in Riyadh, Saudi Arabia, to elucidate the significance of evaluating the burden experienced by caregivers of individuals undergoing PD and HD. The assessment included the caregiver's health, engagement in activities, social interactions, and psychological well-being. Moreover, a comprehensive understanding of the limitations faced by caregivers in terms of their quality of life (QOL) was achieved.

## Materials and methods

Study design

This cross-sectional study was conducted at King Fahad Medical City in Riyadh, Saudi Arabia, to investigate the caregiver population from 15th November 2022 to 1st July 2023.

Sample population and recruitment

The research was centered on caregivers responsible for providing care to patients undergoing dialysis. The study sample was recruited from a medical facility by distributing a voluntary questionnaire to potential participants. The study included caregivers of both genders. The study only included patients who granted their consent. Individuals aged below 18 and responsible for providing care were excluded from the study.

A data collector administered the questionnaire to all caregivers included in the sample. The questionnaire was constructed to employ easily comprehensible language, facilitating effective communication with the participants. This approach aimed to streamline the process of gathering data from caregivers to enable convenience and easy accessibility within the medical facility environment.

Data collection instrument

The researchers used the Zarit Burden Interview Arabic Abridged version (ZBI-A) for this objective. The simplified form of the Zarit Burden Interview (ZBI-A) comprised 12 items, providing a succinct means of assessing the burden experienced by caregivers [17]. Caregivers were asked to assign a rating ranging from 0 to 4 to each item, denoting the frequency or intensity of their caregiving-related experiences. Consistent with the comprehensive ZBI scale, an elevated ZBI-12 score indicates an increased degree of caregiver burden. The scale enables a prompt evaluation and offers significant insights into the caregiver's perceived burden, facilitating expedient data collection and analysis [18].

Data collection

A data collector gave the participants a questionnaire. Each caregiver's responses were measured and assigned a score on a scale ranging from 0 to 48. The data analysis involved calculating these scores to examine the distribution of results. The survey comprised a comprehensive set of questions that evaluated different dimensions of caregiver burden, including its effects on familial and social dynamics, psychological state, capacity to handle obligations, caregiver's physical health, and the relationships between dialysis patients and their caregivers.

Measurements

The variables were assessed using a scale that measured each variable on a range of values from "never" to "nearly always." Subsequently, a score was allocated to each participant's response in accordance with their selected option. The scoring system was specifically developed to enhance the data analysis process, and these scores were computed within the data analysis stage. The co-investigators were responsible for procuring the required information from the participants to ensure the precision of data collection. The researchers administered the questionnaire, meticulously documented the participants' responses, and diligently verified the integrity and precision of the data entry process. The primary objective of this collaborative endeavor between the researchers and co-investigators was to uphold the integrity and rigor of the data throughout the study.

Statistical analysis

Data analysis in this study was conducted using IBM SPSS version 23. The study variables were characterized using descriptive statistics, which included frequencies and percentages for categorical variables and means and standard deviations for continuous variables. The scoring system used in this study was the Short Form Zarit Burden Interview (ZBI-12). This involved aggregating scores from the 12 items, each with a potential range of 0 to 4 points, resulting in a total score ranging from 0 to 48. The ZBI-12 score was interpreted as follows: scores ranging from 0 to 10 indicated the absence or presence of mild burdens, scores ranging from 10 to 20 indicated mild to moderate burdens, and scores exceeding 20 indicated high burdens. The chi-square test was used to examine the association between categorical variables.

Furthermore, a reliability analysis was performed using Cronbach's alpha to evaluate the measurement characteristics of the scales and the mean inter-item correlation. The tests assumed that the data followed a normal distribution. A significance level of p < 0.05 was selected as the threshold for rejecting the null hypothesis, indicating statistically significant results.

Ethical approval

The ethical standards established by the institutional and national research committees, the 1964 Helsinki Declaration and its associated regulations, or comparable ethical principles were followed in this cross-sectional study involving human subjects. The Human Investigation Committee (IRB) of King Fahad Medical City, OHRP/NIH, USA, IRB 00010471, approved this study [Log Number 22-285].

## Results

A total of 137 caregivers were approached, and only 73 agreed to participate. However, only 50 questionnaires were completed. The response rate was 68.49%. Out of 50 participants, the majority were aged 31 to 50 (n = 28, 56%), female (n = 37, 74%), married (n = 26, 52%), having university educations (n = 22, 44%), and employed (n = 21, 42%). In the current study, 51% (n = 25%) were under dialysis for up to 1 year, and 36.7% (n = 18) were under dialysis for 1-5 years. Data regarding the dialysis duration of one individual was missing (Table 1).

**Table 1:** Demographic characteristics of the caregivers

Demographics		Count	%
Total		50	100.0
Age	18–30	17	34.0
	31–50	28	56.0
	51–70	5	10.0
Gender	Male	13	26.0
	Female	37	74.0
Marital status	Married	26	52.0
	Single	20	40.0
	Widowed	2	4.0
	Divorced	2	4.0
Education	None	3	6.0
	Elementary school	9	18.0
	Middle school	3	6.0
	Secondary school	10	20.0
	University	22	44.0
	Postgraduate	3	6.0
Employment	Employed	21	47.7
	Unemployed	20	45.5
	Retired	1	2.3
	Disabled	2	4.5
	Missing	6	
Income	<5000	24	52.2
	5000–10000	11	23.9
	10000–15000	7	15.2
	>15000	4	8.7
	Missing	4	
Years on dialysis	0–1 years	25	51.0
	1–5 years	18	36.7
	5–10 years	4	8.2
	>10 years	2	4.1
	Missing	1	
Caregiver	PD	25	50.0
	HD	25	50.0

The PD and HD caregivers showed no significant differences in gender (p = 0.333), age (0.818), marital status (p = 0.702), employment (p = 0.324), or income between the two groups (p = 0.586). Similarly, no statistically significant difference was observed in the distribution of years on dialysis between the two groups (p = 0.075). In terms of education, it was observed that PD caregivers exhibited a higher percentage of individuals with a university-level education (63.6%) compared to HD caregivers (36.4%, p = 0.012; Table 2).university-level education (63.6%) in comparison to HD caregivers (36.4%). (p = 0.012) (Table 2).

**Table 2:** Distribution of variables among the caregivers

Variables		Total	Caregiver		p-value
			PD	HD	
Total		50	25 (50.0%)	25 (50.0%)	-
Age	18–30	17	8 (47.1%)	9 (52.9%)	0.818
	31–50	28	15 (53.6%)	13 (46.4%)	
	51–70	5	2 (40.0%)	3 (60.0%)	
Gender	Male	13	8 (61.5%)	5 (38.5%)	0.333
	Female	37	17 (45.9%)	20 (54.1%)	
Marital status	Married	26	15 (57.7%)	11 (42.3%)	0.702
	Single	20	8 (40.0%)	12 (60.0%)	
	Widowed	2	1 (50.0%)	1 (50.0%)	
	Divorced	2	1 (50.0%)	1 (50.0%)	
Education	None	3	1 (33.3%)	2 (66.7%)	0.012a
	Elementary school	9	0 (0.0%)	9 (100.0%)	
	Middle school	3	1 (33.3%)	2 (66.7%)	
	Secondary school	10	6 (60.0%)	4 (40.0%)	
	University	22	14 (63.6%)	8 (36.4%)	
	Postgraduate	3	3 (100.0%)	0 (0.0%)	
Employment	Employed	21	12 (57.1%)	9 (42.9%)	0.324
	Unemployed	20	12 (60.0%)	8 (40.0%)	
	Retired	1	1 (100.0%)	0 (0.0%)	
	Disabled	2	0 (0.0%)	2 (100.0%)	
Income	<5000	24	13 (54.2%)	11 (45.8%)	0.586
	5000–10000	11	4 (36.4%)	7 (63.6%)	
	10000–15000	7	2 (28.6%)	5 (71.4%)	
	>15000	4	2 (50.0%)	2 (50.0%)	
Years on dialysis	0–1 years	25	14 (56.0%)	11 (44.0%)	0.075
	1–5 years	18	5 (27.8%)	13 (72.2%)	
	5–10 years	4	3 (75.0%)	1 (25.0%)	
	>10 years	2	2 (100.0%)	0 (0.0%)	
Zarit Burden Interview (ZBI-12)	No to mild burden	23	15 (65.2%)	8 (34.8%)	0.139
	Mild to moderate burden	19	7 (36.8%)	12 (63.2%)	
	High burden	8	3 (37.5%)	5 (62.5%)	
asignificant using chi-square test at <0.05 level					

The reliability statistics provided in Table 3 demonstrate the internal consistency of the ZBI-12 scale, comprising a total of 12 items. Cronbach's alpha, a statistical measure assessing the degree of interrelatedness among items within a scale, was 0.664. The obtained value indicates a moderate degree of internal consistency for the ZBI-12 scale.

**Table 3:** Reliability statistics of scale

Reliability Statistics	Cronbach’s Alpha	N of Items
Zarit Burden Interview (ZBI-12)	0.664	12

The maximum mean score was obtained from Item 11, "You should you be doing more for your relative?" and the least mean score was obtained from Item 3, "Are you angry when you are around your relative?" which was 2.48 ± 1.6 and 0.22 ± 0.6 (Table 4).

**Table 4:** Table 4

Zarit Burden Interview (ZBI-12)	N	Min	Max	Mean	SD
Does the time you spend with your relative mean you don’t have enough time for yourself?	50	0	4	1.56	1.6
Are you stressed due to caring for your relative and trying to meet other responsibilities (work/family)?	50	0	4	1.48	1.5
Are you angry when you are around your relative?	50	0	2	0.22	0.6
Does your relative currently affect your relationship with family members or friends in a negative way?	50	0	4	0.40	1.1
Are you strained when you are around your relative?	50	0	3	0.48	1.0
Has your health suffered because of your involvement with your relative?	50	0	3	0.28	0.7
Do you have less privacy than you would like because of your relative?	50	0	4	0.76	1.4
Has your social life suffered because you are caring for your relative?	50	0	4	0.82	1.4
Have you lost control of your life since your relative’s illness?	50	0	4	0.42	1.0
Are you uncertain about what to do about your relative?	50	0	4	0.98	1.5
Should you be doing more for your relative?	50	0	4	2.48	1.6
Could you do a better job in caring for your relative?	50	0	4	2.34	1.6

The mean ZBI-12 score was 12.22 ± 7.2, suggesting a moderate degree of dispersion among the scores. The analysis of the ZBI-12 scores demonstrates that within the entire sample, 46.0% (n = 23) of participants indicated negligible to minimal burden, 38.0% (n = 19) reported a moderate level of burden, and 16.0% (n = 8) reported a significant level of burden (Table 5).

**Table 5:** Caregivers' response according to ZBI-12 Interview questions

Variables	N	Min	Max	Mean	SD
Zarit Burden Interview (ZBI-12)	50	0	28	12.22	7.2
		Count		%	
Total		50		100.0	
Zarit Burden Interview (ZBI-12)	No to mild burden	23		46.0	
	Mild to moderate burden	19		38.0	
	High burden	8		16.0	

**Figure 1:**
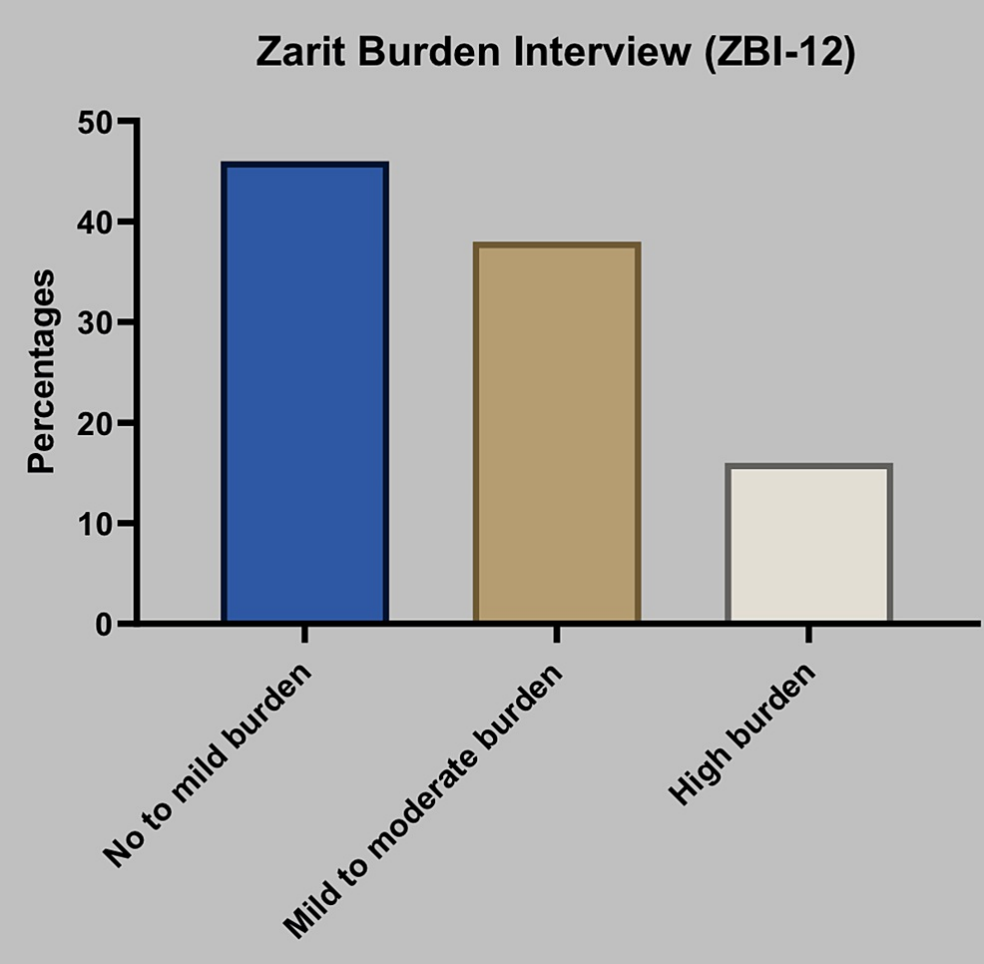
Graphical representation of caregivers’ responses

The results of this study indicate that a significant proportion of the caregivers involved experienced a certain degree of burden, with the majority falling within the classifications of no to mild and mild to moderate burden (Figure 1)

**Figure 2:**
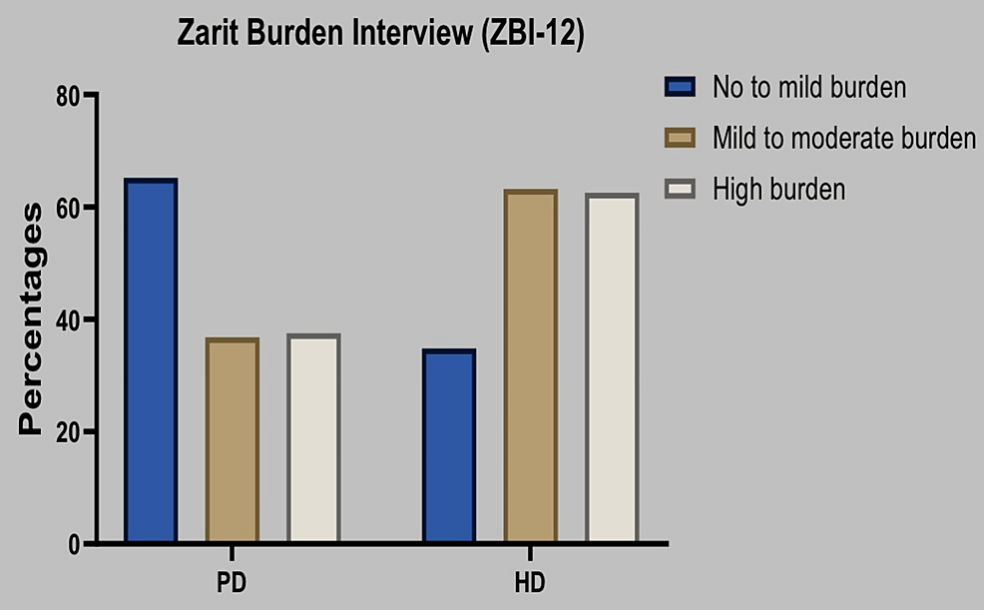
Caregivers' response regarding burden among both the groups

The caregiver burden was assessed as a no to mild level by 46.0% of the overall group, including 65.2% of PD caregivers and 34.8% of HD caregivers. A mild to moderate burden was indicated by 38.0% of the whole group, including 36.8% of PD caregivers and 63.2% of HD caregivers. High levels of burden were indicated by 16.0% of the entire group, including 37.5% of PD caregivers and 62.5% of HD caregivers. Figure 2 represents the caregivers' levels of burden among both groups.

## Discussion

This study aimed to evaluate and contrast the level of burden encountered by caregivers of individuals receiving PD and HD. The findings provided valuable insights into the caregiver burden encountered within these two treatment modalities. This study indicated that most caregivers experienced no to mild or moderate levels of burden for the patients undergoing PD or HD. These results were consistent with a study by Dikmen et al., based on which the caregivers experienced a moderate degree of burden associated with their caregiving responsibilities [19]. Regarding the distribution of burden levels among the groups, a significant proportion of caregivers in the PD and HD groups indicated experiencing either no to mild or mild to moderate burdens. However, a significant proportion of caregivers, especially those in the HD group, encountered a substantial burden. This implies that the act of providing care for individuals undergoing dialysis can be challenging and tiresome, resulting in notable levels of burden experienced by some of the caregivers. According to a study, caregivers of patients undergoing HD experienced moderate caregiving burdens [21].

No statistically significant differences were observed when comparing the levels of burden experienced by caregivers of individuals receiving PD and those of individuals receiving HD. This suggests that PD and HD place a comparable level of responsibility on caregivers. This finding emphasizes the need to provide sufficient support and strategies to effectively address the burden experienced by caregivers across various dialysis modalities. These findings were inconsistent with the findings of other studies. The study conducted by Shimoyama et al. demonstrated a notably low caregiver burden among individuals undergoing PD. The levels of burden experienced by caregivers of patients undergoing PD were found to be significantly lower compared to caregivers of patients undergoing HD, as indicated by a highly significant statistical result (p < 0.005) [21]. Pürlüsoy et al. conducted a study to examine the caregiver burden experienced by individuals caring for patients undergoing HD and PD [22].

The study revealed that caregivers of HD patients reported experiencing moderate to high levels of caregiver .burden, which were comparatively higher than those reported by caregivers of PD patients [23].

Conversely, individuals providing care for people diagnosed with cancer may encounter a significant burden associated with symptom management and adverse consequences of treatment [9, 24]. Managing behavioral concerns and assisting with daily activities pose significant challenges for carers of individuals with dementia [25]. Additionally, individuals providing care for patients with chronic illnesses such as diabetes may encounter carer loads related to managing nutritional and pharmacological needs.

Significant numbers of informal carers for thalassemia and HD patients, 58% and 43%, reported moderate carer loads. Strong correlations existed between carer load and depression (p < 0.0001) and QOL (p < 0.009). Carers of HD patients had higher depression levels than those of thalassemia patients. HD carers had a better QOL than thalassemia carers [26].

The investigation of the demographic variables yielded different patterns. No significant difference in age distribution was found between caregivers of individuals undergoing PD and HD, suggesting that the burden experienced by caregivers is not affected by the caregiver's age. Similarly, the distributions of gender and marital status were similar between the two groups, indicating that these variables may not substantially influence caregiver burden regarding dialysis. According to other studies, the burden experienced by caregivers was significantly influenced by the caregiver's age. Jafari et al. conducted a study yielding comparable findings [26]. Specifically, their research revealed a positive association between the age of caregivers and the extent of caregiving responsibilities. Furthermore, their study identified a noteworthy correlation between caregiver age and the burden they experienced [26].

A cross-sectional study conducted in China revealed moderate levels of caregiver burden, as evidenced by the ZBI score. The statistical analysis results indicated that several factors, including female gender, insufficient financial resources, limited social support, depressive symptoms in patients, and disability, were significant predictors of caregiver burden [27].

The educational backgrounds of PD and HD caregivers exhibited an obvious difference, as a higher percentage of PD caregivers possessed a university-level education compared to HD caregivers. This result implies that individuals with higher education levels may have different views and experiences regarding the burden of caregiving, which their educational history could influence. In the study conducted by Rafati et al. (2020), it was observed that patients with higher education levels undergoing HD therapy were associated with a reduced burden of care [28]. Generally, these findings emphasize the significance of acknowledging and mitigating the challenges faced by caregivers of individuals undergoing dialysis.

In the present investigation, the item with the highest-burden score pertained to the belief that caregivers should undertake additional responsibilities for their family members. The aforementioned observation aligns with the results obtained in prior studies concerning the load experienced by family caregivers in the context of the elderly population. Numerous studies have consistently emphasized that family caring requires a substantial allocation of time, frequently comparable to the responsibilities associated with a part-time occupation. Family caregivers often face a decrease in their work hours, a loss of job benefits, missed career prospects, and, in certain instances, are compelled to withdraw completely from the labor force. The responsibility of providing care can lead to reduced personal and familial leisure time, tension in marital partnerships, and the amplification of financial difficulties [29,30].

Within the context of the current research, it was noted that carers reporting having minimal personal time due to the substantial care they offered to their family exhibited high burden scores. This finding is consistent with other research indicating that caregivers frequently report declining health due to the demands of their caregiving duties [31]. Carers often feel stressed. Mashayekhi et al. found that 72.5% of carers had moderate to severe carer loads [4]. In a separate study by Jafari et al., 37.4% of carers indicated high to very high care loads, while 42.7% reported moderate loads [26]. A recent survey found 72.5% of carers lacked resilience [32]. This shows that these people struggle with emotional problem-solving and coping, indicating the role of resilience in caring and suggesting that building resilience can improve care receivers' experiences. These studies show that carers' emotional and physical health, combined with financial issues, contributes to their burden [32].

A study conducted during the COVID-19 pandemic found no statistically significant differences in QOL, including physical and mental health, between HD and PD carers (p > 0.05). No significant difference in COVID-19 fear was found across the groups (p > 0.05) [33]. However, carers' attitudes and weight greatly impact patient welfare. Regardless of initial enthusiasm and vigor, chronic caregiving can cause physical tiredness and affect mental health [34]. Conversely, health impacts vary. Carers of HD patients feel 27.2% more hardship than those of PD patients (p < 0.001). Care management is 68.4% more difficult for HD carers (p = 0.002). This contradicts a separate study that found HD carers had a 35% burden and PD carers 13% [6]. PD carers had less stress than HD carers in Mexico despite a comparable caregiving load [35].

A study conducted in Riyadh showed that the caregiver burden among PD and HD families ranged from moderate to severe burden. The correlation between caregivers' burden scores and caregivers' education, caregivers' age, and patients' education are negatively correlated only in HD. However, the correlation between caregivers' burden scores and patients' age is significant in both HD and PH [36].

The research emphasizes that the responsibilities associated with caregiving with both PD and HD can impose significant burdens. Therefore, it is crucial to establish suitable support systems and interventions to alleviate the strain experienced by caregivers. Healthcare practitioners should consider personalized support strategies tailored to caregivers' distinct requirements and situations, irrespective of the dialysis modality. Moreover, additional research must be conducted to investigate supplementary variables that could contribute to the burden experienced by caregivers in dialysis settings. These variables include social support, coping strategies, and caregiver resilience.

This study had several limitations. Firstly, the study was conducted in only one center, resulting in a sample of caregivers sharing similar socio-cultural characteristics. Moreover, the study's cross-sectional design limited the ability to adequately assess the existence of causality. The generalizability of the research results for all caregivers of HD and PD patients in Saudi Arabia was limited due to the small sample size and large number of females in the study. Moreover, the study focused on adult patients and may not reflect the opinions of caregivers providing care for pediatric patients undergoing PD or HD.

## Conclusions

This study evaluated and contrasted the burden level encountered by caregivers of individuals receiving PD and HD. The results of the study indicate that caregivers of individuals undergoing PD and HD encounter different degrees of burden, with a significant proportion of caregivers experiencing a substantial burden. However, no statistically significant disparities exist in the levels of burden observed for the two groups. The findings emphasize the importance of providing appropriate resources and measures to effectively address the burden experienced by caregivers in the dialysis context.
